# New Naphthalimide Derivative as a Colorimetric and Fluorescent Probe for Detection of pH, Strong Bases and Volatile Acids

**DOI:** 10.3390/s26082411

**Published:** 2026-04-15

**Authors:** Polya M. Miladinova

**Affiliations:** Organic Synthesis Department, University of Chemical Technology and Metallurgy, 8 Kliment Ohridsky Blvd, 1797 Sofia, Bulgaria; ppolya@uctm.edu

**Keywords:** 1,8-naphthalimide derivative, detection in solution, strip paper and thin film

## Abstract

The development of effective fluorescent probes for the detection of acids and bases, both in solution and in the solid state, is of particular interest worldwide, due to the possibility of preventing hazardous consequences for human health and the environment. In the present work, the synthesis of a 1,8-naphthalimide derivative, designed as a “fluorophore-receptor_1_-spacer-receptor_2_” model, is considered. The compound contains two receptors for analytes in one molecule and can operate as a fluorescent probe via PET and ICT mechanisms. The photophysical behavior of the synthesized derivative in solution, on strip paper, and in thin film was investigated. It was found that the transition from acidic to alkaline medium in solution is associated with a change in color that is visible with the naked eye (yellow–orange-red–blue). The change in fluorescence, both in solution and spread on a supporting surface (strip paper and thin film), can be spectrophotometrically observed. The influence of various volatile acids on the sensing activity of the synthesized compound in solution and deposited on a solid support was investigated. It was found that with increasing acid strength, the fluorescence intensity increases. The strip paper and thin film obtained with the synthesized compound show reversible switching between the “off” and “on” states of fluorescence. The strip paper exhibited good cycling under acid–base vapor stimulation. The results obtained demonstrate the possibility of application of the synthesized compound as a colorimetric and fluorescent probe for determination of pH in solution, and detection of acids, bases, and their vapors in indoor and outdoor residential and industrial premises, as well as in the environment.

## 1. Introduction

Acids and bases are chemical raw materials, widely used in industry and agriculture. The release of acids, bases and their vapors into the environment leads to environmental problems: pollution of water, soil and air. As a result, acid rain can occur, plant and animal environments are threatened, and human life is endangered. The detection of acids and bases using chemical sensors is possible because they convert chemical stimuli into signals that can be easily observed by the naked eye, such as color changes in absorption or fluorescence, or changes in fluorescence intensity observed by spectrophotometers [1,2,3,4,5,6,7].

Pollution of the environment with acids and bases and their vapors and gasses leads to a change in the pH of waters and soils. The research, monitoring and, in many cases, maintaining of pH have led scientists from all over the world to focus their attention exactly on this important parameter [8,9,10,11,12,13].

Among the three macro areas (optical, electrochemical and field effect), where pH sensors can be grouped, optical sensors occupy an important place and play a key role in a number of technologies and human life, because they have good photo- and chemostability [14]. Optical pH sensors are popular in different areas—for measurements of biological tissues or cell cultures; for colorimetric or photometric analyses for safety and control of food, cosmetic products, and drugs; and for monitoring water, soil and air [9,15,16].

Fluorescent pH probes use dye molecules whose fluorescent properties (intensity, wavelength and life time) change predictably as a result of pH variations, often through mechanisms like fluorescence quenching or enhancement. For this purpose, the design of fluorescent probes mainly involves and exploits some phenomena: intramolecular charge transfer (ICT), photoinduced electron transfer (PET), etc. [17].

PET is an important design strategy for the development of “off–on” or “on–off” fluorescent probes. A typical PET-based fluorescent probe uses the “fluorophore–spacer–receptor” format. During the recognition process, the receptor binds to an analyte and either PET is restricted and the fluorescence is turned-on (“off-on”), or PET is activated and the fluorescence is turned-off (“on-off”) [18].

ICT involves the transfer of charge from an electron-rich donor moiety to an electron-poor acceptor moiety, located in the same molecule. If a guest-analyte is near to the donor or acceptor moiety, this will change the photophysical properties of the fluorophore because it will affect the efficiency of ICT. So, in contrast to PET, in ICT, the receptor is directly attached to the donor or acceptor part. Therefore, recognition of the analyte is associated with a change in wavelength of absorption of the fluorophore [19].

A significant success in the detection of various analytes is the development of polymer dye sensors or those immobilized on various surfaces. Commonly, the dyes which can be used in pH sensors usually contain carboxylic or amine groups. The latter can be protonated, or respectively deprotonated, depending on the pH. As a result, the protonated and deprotonated forms display different absorption spectra and one of the two forms is charged and the polarity of the dye changes significantly [20]. This makes it difficult to obtain covalently bound fluorophores in polymers by polycondensation. To produce polymer sensors through copolymerization, a fluorophore containing a polymerizing group and additional time for copolymerization with traditional monomers is required [21,22,23,24]. The possibility of obtaining pH sensors by immobilization of a dye molecule on the polymer [25,26,27,28,29], or applying the fluorophore in the form of a powder or thin film on a different solid surface, is a modern method of obtaining solid-state fluorescence-sensing materials [6,30,31,32].

Among the commonly used dyes for sensors and fluorescent probes, naphthalimide derivatives have an important place as signal elements because of their advantages, including strong fluorescence, large Stokes shifts, and excellent photostability. They have applications in different industrial processes, environmental monitoring, analytical chemistry, medical diagnostics and biological imaging [6,24,25,31,32,33,34,35,36,37,38,39,40]. It is known that in most 1,8-naphthalimide sensors, the C-4 position is responsible for sensor activity, because this position contains the exact receptor fragment that can react with different analytes, which determines the change in color or intensity of the absorption and fluorescence [41,42,43].

**Scheme 1:**
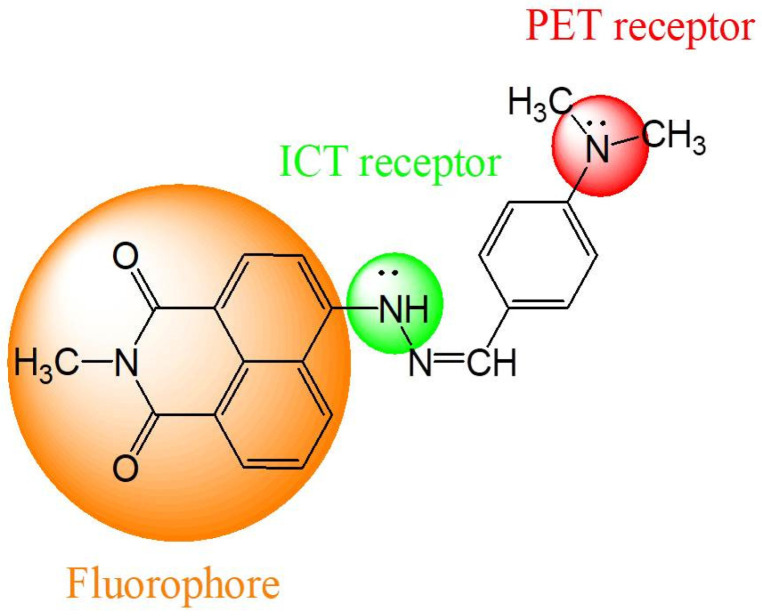
Chemical structure of 1,8-naphthalimide-based fluorescent probe (**5**).

The aim of this work is to synthesize a new derivative of 1,8-naphthalimide, containing two receptors for analytes in one molecule, which can operate as a fluorescent probe via PET and ICT mechanisms (Scheme 1). The target compound is designed to be in the “off state” (quenched fluorescence) due to the presence of PET from the tertiary nitrogen atom from the N,N-dimethylamine group in the side chain of the molecule. Upon encountering an analyte, the PET is expected to be interrupted and the molecule is expected to transition to the “on state” (appearance of fluorescence). At the same time, in the presence of an analyte, the molecule may undergo a hypso- or bathochromic color shift as a result of the reduction or enhancement in the efficiency of the ICT from the donor moiety (the secondary nitrogen atom from the imino group at the C-4 position of the naphthalimide) to the acceptor moiety (the carbonyl group of the naphthalimide).

This work also reports on the behavior of strip paper and thin film of this derivative towards bases, acids and their vapors.

## 2. Materials and Methods

4-bromo-1,8-naphthalic anhydride (**1**), methylamine, 4-(dimethylamino)benzaldehyde (Sigma-Aldrich Co., St. Louis, MO, USA) and hydrazine hydrate (99%) (Fisher Scientific, Waltham, MA, USA) were used. The solvents used in the synthetic procedures, in the photophysical investigation, and the different acids were acetic acid (CH_3_COOH), formic acid (HCOOH), phosphorus acid (H_3_PO_4_), nitric acid (HNO_3_), sulfuric acid (H_2_SO_4_), hydrochloric acid (HCl), ammonia and n-butylamine (Sigma-Aldrich Co., Ltd., St. Louis, MO, USA and Fisher Scientific, Waltham, MA, USA), which were pure or of spectroscopy grade.

The melting point was recorded on a Büchi 535 apparatus (BÜCHI Labortechnik AG, Flawil, Switzerland). Thin-layer chromatography (TLC) was performed on a silica gel plate (Merck, 60 F 254, 20 × 20 cm, 0.2 mm thickness). UV/Vis absorption spectra were recorded on a DLAB SP-UV1100 spectrophotometer (DLAB Scientific Co., Ltd., Beijing, China) and IR spectra were recorded on a Nicolet iS50 FTIR-ATR (Thermo Scientific, Madison, WI, USA). ^1^H NMR and ^13^C NMR analyses were performed on a Bruker AV-600 spectrometer (BRUKER AVANCE II+ 600 MHz, Bruker, Billerica, MA, USA) using dimethyl sulfoxide-d6 as a solvent. Fluorescence spectra were recorded on spectrometer FS-2 (Scinco, Seoul, Republic of Korea). High-resolution mass spectra (HRMS) were recorded on a Q Exactive Plus Hybrid Quadrupole-Orbitrap Mass Spectrometer (Thermo Fisher Scientific Inc., Waltham, MA, USA, HESI HRMS) in positive or negative mode.

The Stokes shift values (ν_A_ − ν_F_) were calculated by Equation (1), where λ_A_ and λ_F_ are wavelengths of absorption and fluorescence, respectively.

(1) $${{(\nu }_{A}-\nu }_{F})=\left(\frac{1}{\lambda_{A}}-\frac{1}{\lambda_{F}}\right)\times 10^{7}$$

The fluorescence quantum yield (*Φ*_F_) was calculated using Coumarin 6 (*Φ*_F_ = 0.78 in ethanol) as standard on the basis of the absorption and fluorescence spectra by Equation (2), where A_ref_, S_ref_, n_ref_ and A_sample_, S_sample_, n_sample_ stand for the absorbance at the exited wavelength, the integrated emission band area and the solvent refractive index of the standard and the sample, respectively [44].

(2) $$\Phi_{F}=\Phi_{ref}\left(\frac{S_{sample}}{S_{ref}}\right)\left(\frac{A_{ref}}{A_{sample}}\right)\left(\frac{n_{sample}^{2}}{n_{ref}^{2}}\right)$$

A pH meter Metrohm 704 (Metrohm AG, Herisau, Switzerland) coupled with a combined pH electrode for the pH measurement was used. The commercial standard buffers for pH 2, 7 and 10 (Sigma-Aldrich Co., St. Louis, MO, USA) were used for calibration.

The pKa values were calculated using Equation (3), where IFmax and IFmin are respectively the maximum and minimum fluorescence intensity in the pH range studied, and IF is the fluorescence intensity at the corresponding pH [45]:

(3) $${pK}_{a}\;=pH-\log\left[\frac{\left(IFmax-IF\right)}{\left(IF-IFmin\right)}\right]$$

For the preparation of impregnation with the target compound (**5**) strip paper, filter paper was soaked in solution of compound (**5**) in ethanol with a concentration of 1 × 10^−2^ mg/mL.

The thin film of compound (**5**) was prepared on glass support after the application of its chloroform-saturated solution and consequent evaporation of the solvent.

### 2.1. Synthetic Procedure

The synthesis of 4-bromo-N-methyl-1,8-naphthalimide (**2**) was accomplished according to a method described previously [46]. The data corresponds with the one in the literature.

#### 2.1.1. Synthesis of 4-Hydrazinyl-N-methyl-1,8-naphthalimide (**3**)

To a solution of 4-bromo-N-methyl-1,8-naphthalic anhydride (**2**) (1.45 g, 0.005 mol) in 100 mL of ethanol, 0.50 g (0.01 mol) of hydrazine monohydrate was added. The reaction mixture was refluxed for 4 h under stirring (monitored by TLC, n-heptane/acetone (1:1 *v*/*v*) eluent system) and then poured into water. The precipitate was collected by filtration, washed with water, and dried. The product (**3**) was an orange solid (3.38 g, 97%), mp: 204–206 °C, R_f_ 0.52, λ_max_^abs^ (DMF) = 448 nm, λ_max_^fl^ (DMF) = 517.8 nm, *Φ*_F_ = 0.008. IR, cm^−1^: 3339 and 3239 (νNH_2_), 3100 (νCH_3_), 3050 (ν–CH), 1696 and 1652 (ν-C=O), 1569 (δNH), 1536 (δNH), 1396 and 1362 (δCH_3_), 1236 (νC–N), 968 (ArH) (Figure S1). ^1^H NMR (dimethyl sulfoxide, δ/ppm): 8.57–8.56 (d, 1H, ArH), 8.53–8.50 (m, 1H, ArH), 8.34–8.32 (d, 1H, ArH), 8.21–8.20 (d, 1H, ArH), 8.02–7.96 (t, 1H, ArH), 7.24–7.23 (ds, 1H, -NH-), 5.79 (bs, 2H, -NH_2_), 3.38 (s, 3H, -CH_3_) (Figure S2). ^13^C NMR (dimethyl sulfoxide, δ/ppm): 160.50, 160.46, 133.23, 132.10, 131.90, 131.47, 129.35, 104.61 (Figure S3). HRMS: Mm_exact_ [g/mol] calcul. for C_13_H_11_N_3_O_2_—241.09, observed 241.07312; [M+H]^+^ observed 242.07642; [M+H]^−^ observed 240.07794 (Figure S4).

#### 2.1.2. Synthesis of 6-(2-(4-(Dimethylamino)benzylidene)hydrazinyl)-2-methyl-1H-benzo[de]isoquinoline-1,3(2H)-dione (**5**)

To a solution of 4-hydrazinyl-N-methyl-1,8-naphthalimide (**3**) (0.24 g, 0.001 mol) in 5 mL of absolute ethanol, 4-(dimethylamino)benzaldehyde (**4**) (0.15 g, 0.001 mol) and a drop of glacial acetic acid as catalyst were added. The reaction mixture was refluxed for 5 h under stirring (monitored by TLC, n-heptane/acetone (1:1 *v*/*v*) eluent system). The reaction mixture was cooled, resulting in a precipitate. The filtrate was evaporated in a vacuum evaporator. TLC showed that both the precipitate and the filtrate contained the target product. The product obtained was recrystallized from ethanol/acetone (2:1). Dark red crystals with a melting point of 233–235 °C were obtained. Yield 0.33 g (88%). R_f_ 0.40, λ_max_^abs^ (DMF) = 488 nm. IR, cm^−1^: 3340 (νNH), 2895 (νCH_3_), 2810 (ν–CH), 1649 (ν-C=O), 1612 (ν-CH=N), 1570 (δNH), 1529 (δ-CH=N-), 1337 (δCH_3_), 1234 (νC–N), 1179 (νC–N), 946, 814 and 765 (ArH) (Figure S5). ^1^H NMR (dimethyl sulfoxide, δ/ppm): 8.64–8.60 (t, 1H, ArH), 8.48 (s, 1H, =CH-), 8.41–8.38 (dd, 2H, ArH), 8.29–8.25 (d, 1H, ArH), 7.80–7.78 (d, 2H, ArH), 7.69–7.66 (dd, 1H, ArH), 6.88–6.86 (d, 2H, ArH), 6.84–6.82 (ds, 1H, -NH-), 3.10 (s, 6H, 2 × -CH_3_), 3.04 (s, 3H, -CH_3_) (Figure S6). ^13^C NMR (dimethyl sulfoxide, δ/ppm): 171.75, 160.45, 153.48, 134.40, 133.18, 132.15, 131.78, 131.46, 130.93, 130.68, 129.20, 128.65, 123.81, 112.35, 111.85 (Figure S7). HRMS: Mm_exact_ [g/mol] calcul. for C_22_H_20_N_4_O_2_—374.16, observed 374.16077; [M+H]^+^ observed 375.16440; [M+H]^−^ observed 373.16571 (Figure S8).

## 3. Results

### 3.1. Design and Synthesis

The new compound (**5**) was designed as a fluorophore with two receptors for analytes (Scheme 1).

**Scheme 2:**
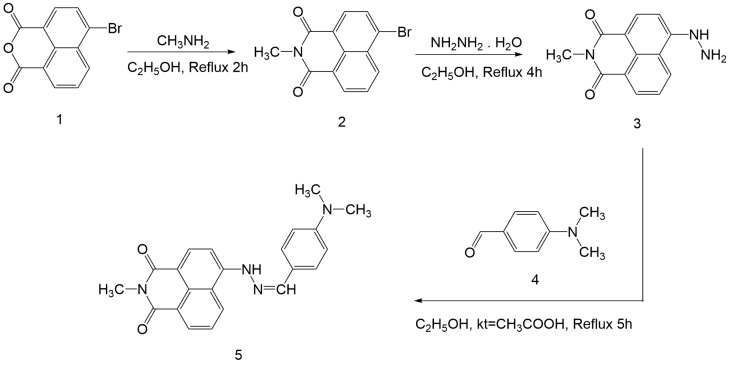
Synthesis of 1,8-naphthalimide derivative (**5**).

The synthesis of desired compound (**5**) was performed in three steps according to Scheme 2.

**Figure 1:**
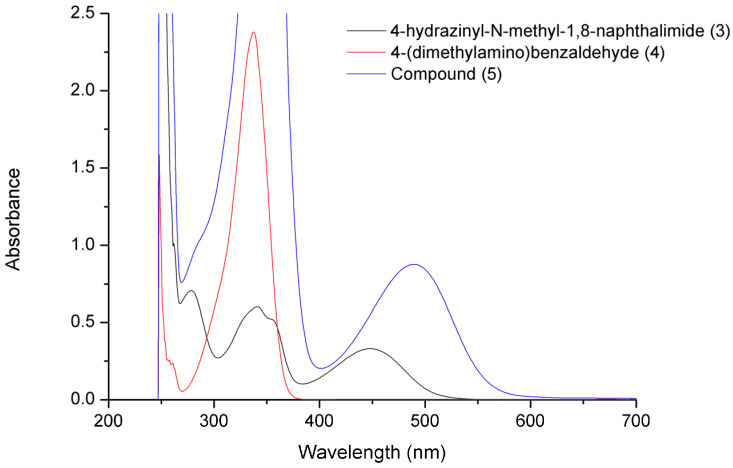
Absorption spectra of 4-hydrazinyl-N-methyl-1,8-naphthalimide (**3**), 4-(dimethylamino)benzaldehyde (**4**) and compound (**5**) in solution of DMF.

Compound (**5**) was isolated and characterized by TLC, melting point, UV/Vis, IR, ^1^H NMR, ^13^C NMR and HRMS. From the absorption spectra obtained for target compound (**5**) and compounds (**3**) and (**4**), used directly for its synthesis (Figure 1), it is seen that compound (**3**) absorbs in the visible region with λ_max_^abs^ = 448 nm, while compound (**4**) absorbs in the UV region with λ_max_^abs^ = 338 nm. The maximal wavelength of target compound (**5**) was bathochromically shifted (λ_max_^abs^ = 488 nm) due to an increase in the donor ability of the nitrogen atom in the C-4 position of the naphthalimide ring.

### 3.2. Chemosensing Properties of Compound (***5***) in Solution

#### 3.2.1. Photophysical Characteristics of Compound (**5**)

The basic photophysical characteristics of compound (**5**) were studied in solvents of different polarity (Table 1). The absorption of the compound showed a bathochromic shift with increasing solvent polarity, which is common for this class of ICT fluorophores. A small fluorescence and small quantum yield (*Φ*_F_) were observed in the solvents with small polarity, as well as an absence of fluorescence in the solvents with high polarity.

The Stokes shift is an important parameter, which indicates the difference between the structure and functional properties of the 1,8-naphtalimide luminophores in their exited and ground states. The obtained results show that the polarity of the organic solvents has a small effect on the Stokes shift values.

#### 3.2.2. Influence of pH on the Absorption Properties of Compound (**5**)

**Figure 2:**
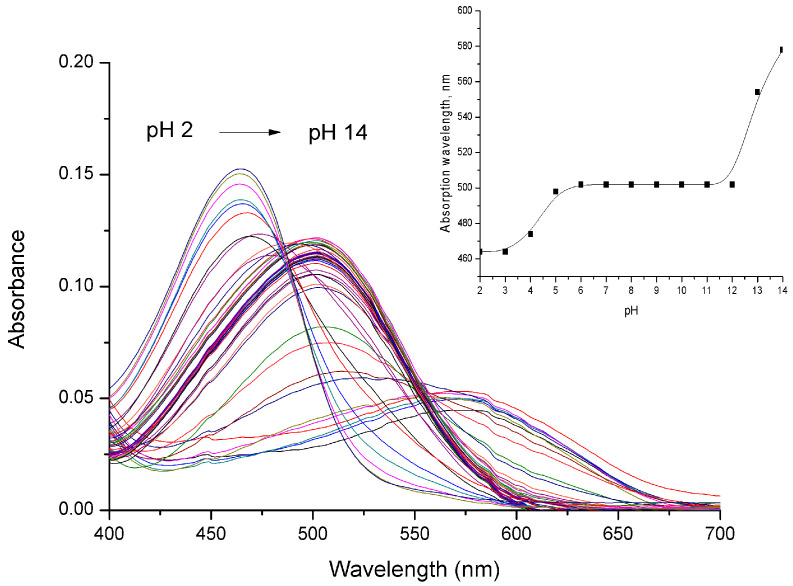
Absorption spectra of compound (**5**) in water/DMF solution (1:1, *v*/*v*) at different pH. Inset: Maximal absorption wavelength of compound (**5**) at different pH.

The photophysical behavior of compound (**5**) as a function of pH was investigated in a water/DMF solution (1:1, *v*/*v*). Using the titration method with small amounts of dilute hydrochloric acid, the absorption spectra of compound (**5**) were recorded at different pH in the pH range of 2–14. Data are given in Figure 2. We can see that in the range of pH 5.5–12, λ_max_^abs^ is 502 nm. In acidic medium (pH < 5.5), as a result of protonation of the secondary nitrogen atom in the C-4 position of naphthalimide, a hypsochromic shift in λ_max_^abs^—from 502 nm to 464 nm—occurs. In strong alkaline medium (pH > 12), as a result of deprotonation of the same nitrogen atom, λ_max_^abs^ shifts bathochromically (from 502 to 578 nm) (Figure 2, inset).

**Figure 3:**
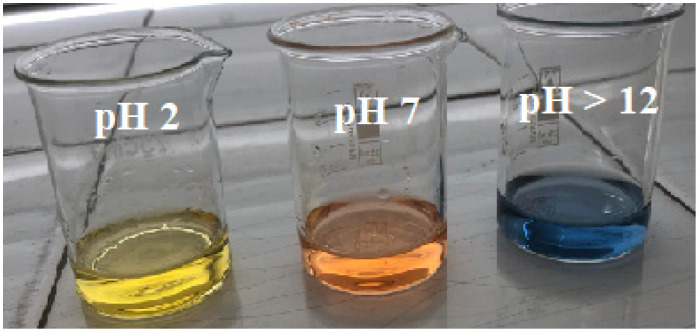
Solutions of compound (**5**) in water/DMF (1:1, *v*/*v*) at different pH.

The change in the absorption of the compound’s solution can also be observed with the naked eye—the color of the solution changes from yellow through orange—to be red to blue from an acidic to alkaline media (Figure 3).

#### 3.2.3. Influence of pH on the Fluorescence Properties of Compound (**5**) in Solution

**Figure 4:**
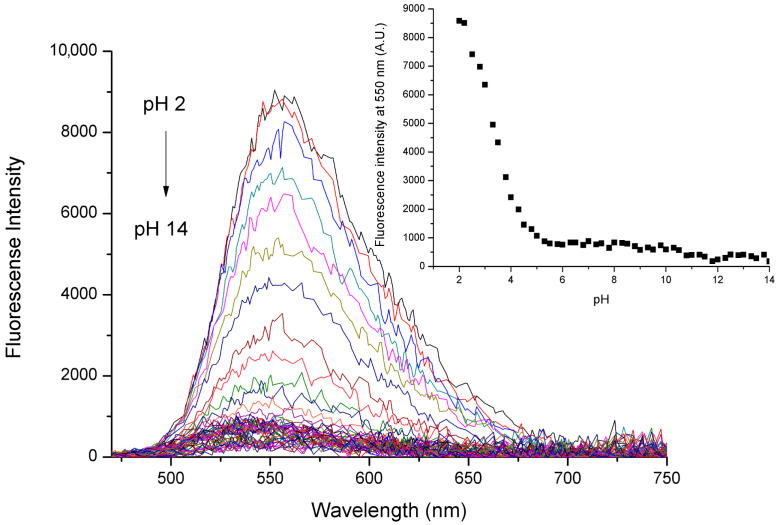
Fluorescence spectra (λ_ex_ = 460 nm, 700 V) of compound (**5**) in water/DMF (1:1, *v*/*v*) solution at different pH. Inset: Titration curve of compound (**5**) at 550 nm.

Emission characteristics of compound (**5**) were investigated in a water/DMF solution (1:1, *v*/*v*) and they showed that they depend on the pH of the medium (Figure 4).

In the range of pH 5.5–14 the fluorescence of compound (**5**) is negligible (the fluorescence intensity is below 1000) and it can be assumed that the compound is in the “off state”. When the medium is acidified (pH < 5.5), an increase in the fluorescence intensity with λ_max_^fl^ 552 nm is observed, and the compound switches to the “on state”.

A fluorescence quenching parameter FQ = I_max_/I_min_ was used for the fluorescence changes of compound (**5**) as a function of pH, where I_max_ is the maximum fluorescence intensity at pH 2 and minimum fluorescence intensity I_min_ at pH 5.5 (FQ = 10.7).

The observed fluorescence changes in the range of pH 2–14 (Figure 4) were analyzed according to Equation (3) [45] and the value of pKa = 3.49 was calculated.

**Figure 5:**
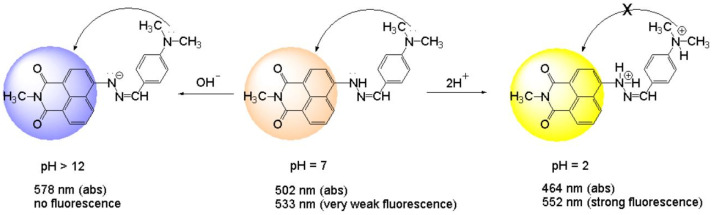
Mechanism of operation of fluorescent probe (**5**).

From the research conducted, the following mechanism of operation of compound (**5**) can be proposed (Figure 5):

#### 3.2.4. Influence of Kind of Acid on the Absorption and Fluorescence Properties of Compound (**5**) in Solution

To understand the sensing effect of compound (**5**) on different volatile acids, some commonly used acids in the laboratory, namely acetic acid (CH_3_COOH), formic acid (HCOOH), phosphorus acid (H_3_PO_4_), nitric acid (HNO_3_), sulfuric acid (H_2_SO_4_), and hydrochloric acid (HCl), were used.

**Figure 6:**
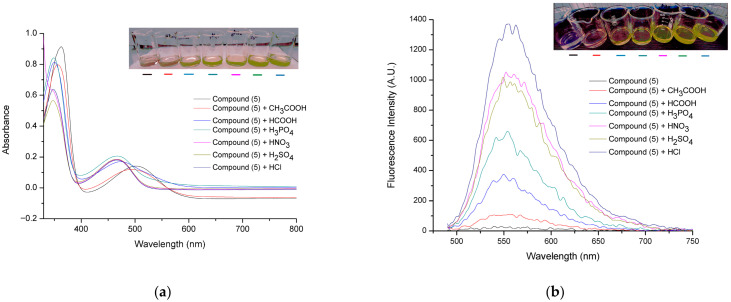
Absorption and fluorescence spectra of the solutions of compound (**5**) in water/DMF (1:1, *v*/*v*) with different acids: (**a**) absorption spectra; (**b**) fluorescence spectra. Inset: Solutions of compound (**5**) with different acids.

For this purpose, to the solution of compound (**5**) in water/DMF (1:1, *v*/*v*) with a concentration of 5 × 10^−3^ mg/mL, 0.1 mL of corresponding acid was added. The absorption and fluorescence spectra of the investigated solutions are given in Figure 6a,b.

### 3.3. Colorimetric and Fluorescence Sensing Investigations on Strip Paper and in Thin Film

Considering the basic researched photophysical properties of compound (**5**) in a solution, the next goal of this work was to investigate its applicability for detection of acids, bases and their vapors using strip paper and thin film. For this purpose, strip paper impregnated with compound (**5**) and thin films on glass support was prepared.

#### 3.3.1. Investigation of the Indicator and Sensory Properties of Compound (**5**) on Strip Paper

**Figure 7:**
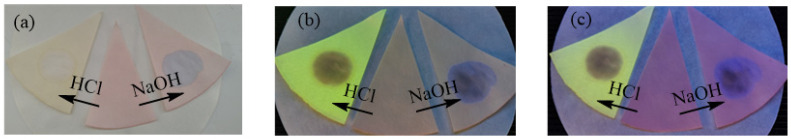
Colorimetric and fluorescence changes of strip paper obtained with compound (**5**) before (middle paper) and after drip of acid or basic solution observed with the naked eye (**a**) and under UV light—254 nm (**b**) and 366 nm (**c**).

**Figure 8:**
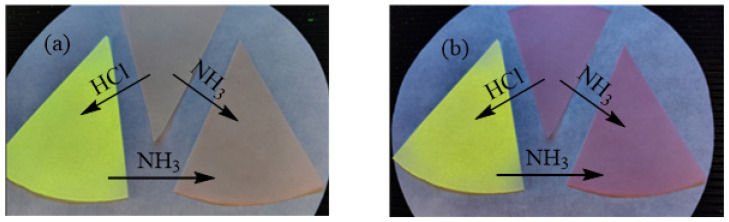
Colorimetric and fluorescence changes of strip paper obtained with compound (**5**) before (middle paper) and upon exposure to vapors of hydrochloric acid or ammonia under UV light—254 nm (**a**) and 366 nm (**b**).

The strip paper obtained with compound (**5**) had an orange-red color and did not have fluorescence (middle paper in Figure 7 and Figure 8). When a drop of hydrochloric acid or sodium hydroxide solution was applied to this paper, a yellow coloration of the whole paper (with hydrochloric acid) or a blue coloration only in the spot (with sodium hydroxide) was observed with the naked eye (Figure 7a).

Under UV light, yellow fluorescence of the paper treated with hydrochloric acid was observed. The paper treated with sodium hydroxide (or potassium hydroxide) did not have fluorescence (Figure 7b,c).

The same effects were observed when the indicator paper was exposed to ammonia and acid vapors (for a maximum of 10 s) (Figure 8). When the acid vapor-treated indicator paper was exposed to ammonia vapors (for a maximum of 10 s again), it restored its original color, and lost its fluorescence.

**Figure 9:**
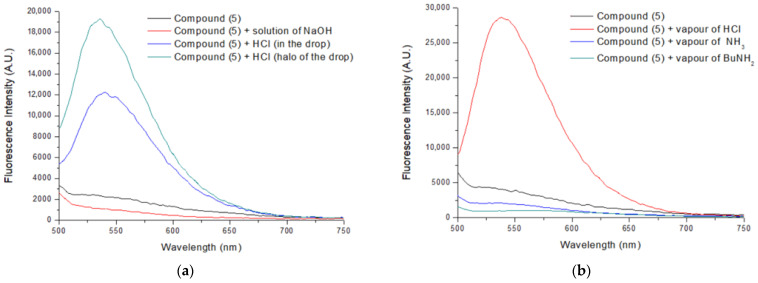
Fluorescence spectra (λ_ex_ = 480 nm, 550 V) of strip paper obtained with compound (**5**): (**a**) before and after drip of acid or basic solution; (**b**) after exposure to the vapors of hydrochloric acid, ammonia and butylamine (BuNH_2_).

The fluorescence spectrum of the indicator paper obtained with compound (**5**) showed that the fluorescence is negligible and therefore considered non-fluorescent. When the acid solution was dropped, the indicator paper showed maximal fluorescent intensity at 535.8 nm and the enhancement in fluorescence was 5 times into the drop and 8 times in its halo in comparison with paper not treated with hydrochloric acid solution. After dropping a solution of sodium hydroxide, the paper did not significantly change its state (i.e., no fluorescence was observed) (Figure 9a).

Similar results were observed when the indicator paper was exposed to the vapors of hydrochloric acid and ammonia. The enhancement in fluorescence during the exposure of acid vapors was 7 times in comparison with paper not treated with vapors (Figure 9b). Except for vapors of ammonia, the indicator paper was exposed to the vapors of butylamine, because it is known that it is more basic than ammonia. The result obtained is comparable to that obtained with the application of ammonia vapors (Figure 9b).

**Figure 10:**
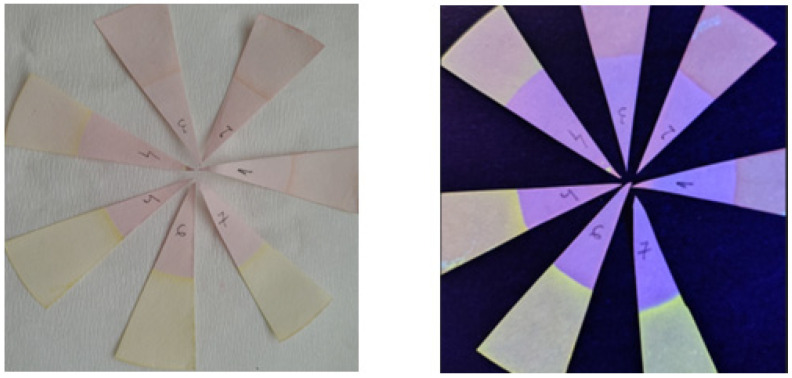
Colorimetric and fluorescence changes of strip paper obtained with compound (**5**) observed with the naked eye (**left**) and under UV light (**right**) before and after drip of different acids (1—indicator paper without acid; 2—with CH_3_COOH; 3—with HCOOH; 4—with H_3_PO_4_; 5—with HNO_3_; 6—with H_2_SO_4_; 7—with HCl).

To understand the sensing effect of strip paper obtained with compound (**5**) on different volatile acids, acetic, formic, phosphorus, nitric and sulfuric acids were used too. When the indicator paper was treated with the acids, a yellow coloration and yellow fluorescence were observed only with strong acids—phosphorus (pK_a_ = 2.16), nitric (pK_a_ = −1.3), sulfuric (pK_a_ = −3) and hydrochloric acid (pK_a_ = −5.9) (Figure 10, number 4–7).

**Figure 11:**
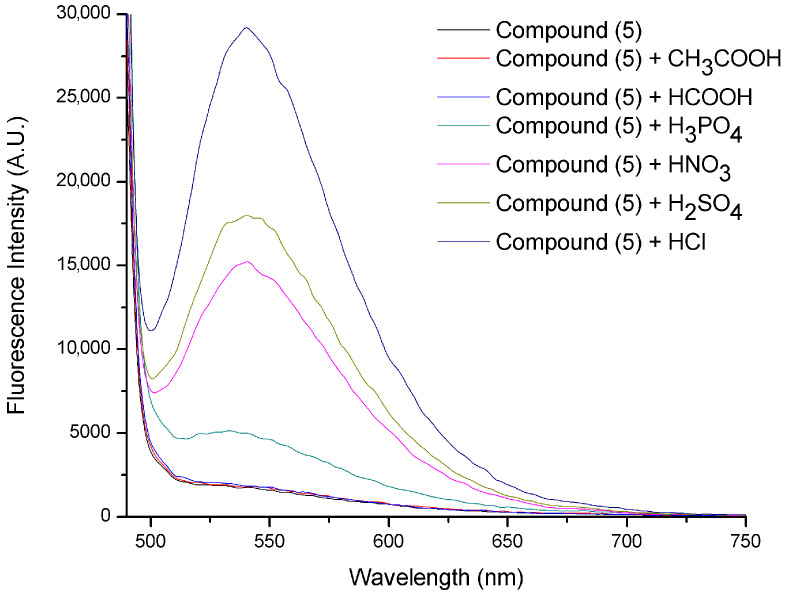
Fluorescence spectra of the strip paper obtained with compound (**5**) treated with vapors of different acids.

The fluorescence spectra of the indicator paper obtained with compound (**5**) after treating with the different acids show that the fluorescence was in the area of 500–650 nm with λ_max_ = 540 nm and enhances with the increase in acid strength (Figure 11). Fluorescence is not observed when weak acids such as acetic (pK_a_ = 4.76) and formic acid (pK_a_ = 3.75) are used, at least with the paper prepared in this way (with this concentration of compound (**5**)).

#### 3.3.2. Acid–Base Vapor Response

**Figure 12:**
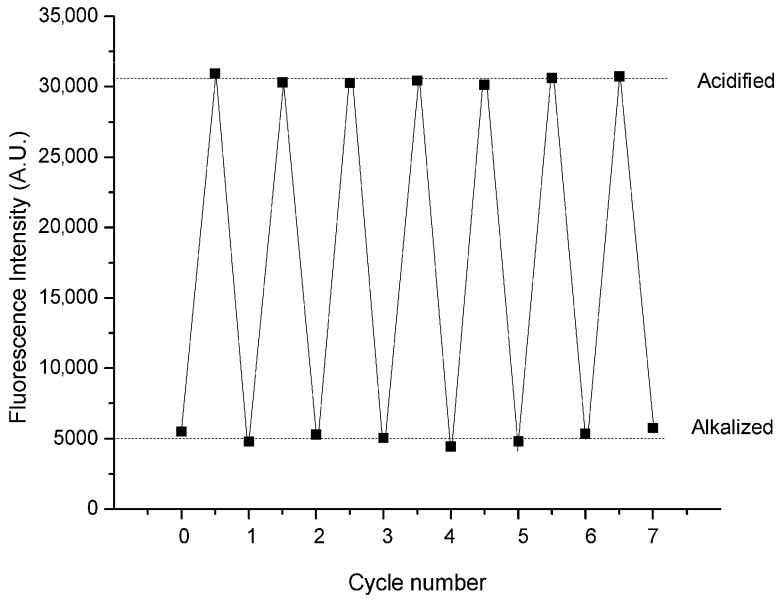
The repeated cycle of the acid–base vapor stimulation process.

From the research conducted and the data presented in Figure 9 and Figure 11, it was observed that the emission properties were altered by acid–base vapors. For the investigation of the possibility of reproducibility of the results when treating the strip paper with acidic and basic vapors, one of the same strip paper was exposed consequently to hydrochloric acid vapors (for 10 s) and ammonia vapors (for 10 s). Using a fluorimeter, the fluorescence intensity value was determined after each treatment with acidic or basic vapors. Under the influence of acidic vapors, the paper was in the “on state”, and under the influence of basic vapors—in the “off state”. Data are given in Figure 12.

#### 3.3.3. Investigation of the Sensory Properties of Compound (**5**) in Thin Film

The thin solid film prepared was exposed to vapors of strong acids (phosphorus, nitric, sulfuric and hydrochloric) and ammonia, and were then subjected to a study to evaluate their photophysical behavior.

**Figure 13:**
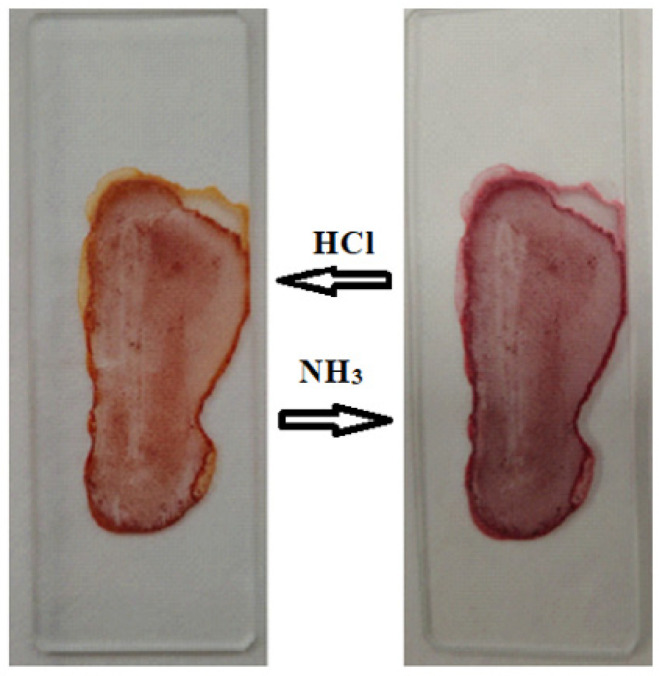
Colorimetric changes in the thin film of compound (**5**) upon exposure to vapors of hydrochloric acid and ammonia.

Compound (**5**) did not have emissive properties in an aggregated state and formed non-fluorescent films with a dark red color (Figure 13, on the right). In the presence of vapors of acids, the thin solid film underwent a color change from dark red to dark orange (Figure 13, on the left), with the presence of fluorescence. When the last one was fumigated with ammonia vapor, its color quickly returned to dark red and the quenching of the fluorescence was observed.

**Figure 14:**
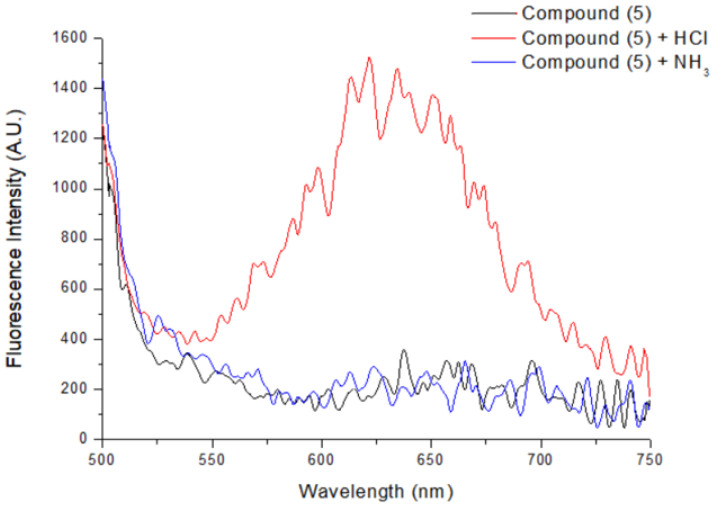
Fluorescence spectra (λ_ex_ = 480 nm, 550 V) of thin film of compound (**5**) before and after exposure to the vapors of hydrochloric acid and ammonia.

The fluorescence spectrum of the thin film obtained with compound (**5**) showed that the fluorescence is negligible and therefore considered non-fluorescent. When the acid vapors were applied, the thin film showed maximal fluorescent intensity at 621.7 nm and the enhancement in fluorescence was 9 times in comparison with thin film not treated with vapors of hydrochloric acid. The same effect was observed during the application of vapors of nitric, sulfuric and phosphoric acid. The fluorescence was practically completely quenched after application of ammonia vapor (Figure 14). An attempt was also made with n-butylamine (pK_b_ = 10.78), known as a stronger base than ammonium hydroxide (pK_b_ = 9.25), and the result was similar.

## 4. Discussion

The new compound (**5**) was designed on the “fluorophore-receptor_1_-spacer-receptor_2_” model (Scheme 1), where 1,8-naphthalimide was chosen as a fluorophore. The secondary nitrogen atom of the imino group located at position C-4 of naphthalimide plays the role of receptor_1_, which operates through the ICT mechanism (ICT receptor). The tertiary nitrogen atom from an N,N-dimethylamine group in the side chain of the molecule plays the role of receptor_2_, which operates through the PET mechanism (PET receptor).

From the studies carried out to determine the photophysical characteristics of compound (**5**), a small fluorescence and small quantum yield (*Φ*_F_) were observed in the solvents with small polarity as a result of the presence of PET from the tertiary nitrogen atom from the N,N-dimethylamine group. With increasing solvent polarity, the absence of fluorescence is observed probably as a result of enhanced PET.

The influence of pH on the absorption properties of compound (**5**) was investigated. From the results obtained, it can be concluded that the protonation of the secondary nitrogen atom of the imino group at the C-4 position of naphthalimide in acid media leads to the decrease in ICT transition in the fluorophore, and a hypsochromic shift is observed in its absorption spectrum. Conversely, the deprotonation of the same nitrogen atom in alkaline media leads to an increase in ICT transition in the fluorophore, and a bathochromic shift is observed (Figure 2). These changes in absorption at different pH are expressed as a color change from yellow, through orange-red to blue, and can be observed with the naked eye (Figure 3).

The results obtained from the absorption behavior of compound (**5**) as a function of pH show its potential as a colorimetric probe for the detection of hydrogen protons and hydroxyl anions in solution, as a result of protonation and deprotonation of an imino group in the C-4 position. This makes the studied compound an effective probe for determination of pH with more sensitivity in the pH ranges of 2–5.5 and 12–14.

The influence of pH on the fluorescence properties of compound (**5**) was investigated. From the results obtained, it can be concluded that in the range of pH 5.5–14, compound (**5**) is in the “off state”. This is a result of the presence of PET between a tertiary nitrogen atom from the N,N-dimethylamine moiety and a naphthalimide fluorophore. When the medium is acidified (pH < 5.5), an increase in the fluorescence intensity is observed. The compound switches to the “on state” as a result of the protonation of the tertiary nitrogen atom of the N,N-dimethylamine moiety and the interruption of the PET (Figure 4).

Considering the above, the mechanism of action of probe (**5**) is shown schematically (Figure 5). It can be seen that in an acidic medium (pH < 5.5), probe (**5**) is in the “on state” and has a yellow color, while in a strongly alkaline medium (pH > 12), it is in the “off state” and has a blue color. In the pH range of 5.5–12, probe (**5**) is in the “off state” and has an orange-red color.

The influence of kind of acid on the absorption and fluorescence properties of compound (**5**) in solution was investigated. The studies show that with the increase in acid strength, the maximum absorption wavelength undergoes a hypsochromic shift (Figure 6a), and the fluorescence intensity enhances (Figure 6b). The studies conducted show the possibility of the synthesized compound to serve as a fluorescent probe for the detection of various volatile acids in solution.

Considering the researched basic photophysical properties of compound (**5**) in solution, the next goal of this work was to investigate its applicability for the detection of acids, bases and their vapors using strip paper and thin film. That is why they were prepared and some investigations were done with them.

The strip paper obtained with compound (**5**) had an orange-red color and did not have fluorescence (Figure 7). After adding a drop of hydrochloric acid to the indicator paper prepared, a yellow coloration and yellow fluorescence were observed not only at the drop site, but also in the whole paper because of the highly volatile vapors of hydrochloric acid (Figure 7a). The change in color is a result of the protonation of the secondary nitrogen atom of the imine group in the C-4 position of the naphthalimide ring, which decreases the fluorophore’s ICT efficiency.

Similar results were observed only with the stronger acid (phosphorus, nitric and sulfuric), but not with weak acids such as acetic and formic acid (Figure 10). Probably under the influence of weak acids such as acetic and formic acid, the ICT receptor does not capture the analytes (no protonation of the secondary nitrogen atom occurs).

From Figure 9a, Figure 10 and Figure 11, it is established that only in the presence of strong acids is a fluorescence of the strip paper observed, which is a result of the protonation of the tertiary nitrogen atom of the N,N-dimethylamine moiety and an interruption of the PET.

After adding a drop of sodium hydroxide solution, blue coloration only at the drop site and the absence of fluorescence were observed (Figure 7). This is a result of the deprotonation of the secondary nitrogen atom of the imine group in the C-4 position of naphthalimide, which enhances the fluorophore’s ICT efficiency and the deprotonation of the tertiary nitrogen atom of the N,N-dimethylamine moiety, which recovers the PET (Figure 7 and Figure 9a). Since potassium and sodium hydroxide are approximately equally basic, a similar result was observed.

Upon exposure of the indicator paper to vapors of the abovementioned acids, similar results were obtained (Figure 9b, Figure 10 and Figure 11). In contrast, when the indicator paper was exposed to vapors of ammonia, a change in the color of the paper and fluorescence was not observed (Figure 8 and Figure 9b). Except for vapors of ammonia, the indicator paper was exposed to the vapors of n-butylamine, because it is known that it is more basic than ammonia (pKb = 10.78 of n-butylamine; pKb = 9.25 of ammonia). The result obtained is comparable to that obtained with the application of ammonia vapor (Figure 9b). The conclusion that can be made is that the basic properties of the ammonia and n-butylamine vapors are sufficient to deprotonate the tertiary nitrogen atom, responsible for fluorescence (no fluorescence), but insufficient to deprotonate the secondary nitrogen atom, responsible for the color change (no change in the color).

When the acid vapor-treated indicator paper is exposed to ammonia vapors, it restores its original color, and loses its fluorescence (Figure 8). This was the basis for investigating the paper’s ability to be cyclic when applying acid–base stimulation. It was found that under the action of acid–base stimulation, the transition from the “off-state” (alkalized) to the “on state” (acidified) can be registered seven times with one of the same strip paper (the “on state” and “off state” had approximately the same value 7 times) (Figure 12).

The results obtained with the strip paper demonstrate the potential of the synthesized compound (**5**) in this form to be an effective probe for detection of vapors of phosphorus, nitric, sulfuric and hydrochloric acids with good cycling under acid–base vapor stimulation. At the same time, the strip paper can be used as an indicator only for detection of solutions of strong bases as sodium and potassium hydroxide.

The thin solid films of compound (**5**), prepared on glass support, had a dark red color (Figure 13). They were exposed to vapors of different acids. The results obtained from the investigations of the sensory properties of probe (**5**) in thin film are similar to those on the strip paper. They demonstrate the potential of compound (**5**) to be a fluorescent probe in this form for the detection of strong acid vapors with cycling under acid–base vapor stimulation.

## 5. Conclusions

A new 1,8-naphthalimide derivative, designed on the “fluorophore-receptor_1_-spacer-receptor_2_” model, was synthesized. The secondary nitrogen atom attached at the C-4 position of naphthalimide and the tertiary nitrogen atom from the N,N-dimethylamine group in the side chain were designed to be receptors for analytes which operate via ICT and PET mechanisms. Using the protonation of the secondary amine, directly attached to the naphthalimide and the tertiary amine in the side chain, in the presence of acids and their vapors, not only in solution, but also in the thin film and strip paper, the compound shows a considerable hypsochromic shift in color and strong fluorescence. At the same time, the process of deprotonation in the presence of ammonia vapors caused notable fluorescence quenching of the compound and bathochromic shift in color. When using different acids and their vapor, as their strength increases, the hypsochromic shifts in absorption and in fluorescence intensity increase, and this gives the possibility of the synthesized compound to serve as a fluorescent probe for detection of various volatile acids in solution. The strip paper and thin film obtained with the synthesized compound show reversible switching between “off” and “on” states of fluorescence. The strip paper and thin film exhibited good cycling under acid–base vapor stimulation, which indicates that they could be used as excellent acid–base-responsive materials. As a result of this investigation, it can be concluded that the synthesized compound has high potential as a colorimetric and fluorescent probe for determination of pH, and for detection of strong bases and volatile acids.

## Figures and Tables

**Table 1:** Basic photophysical characteristics of compound (**5**) in solvents of different polarity.

Solvent	λ^abs^, nm	λ^fl^, nm	ν_A_ − ν_F_, cm^−1^	*Φ*_F_
Chloroform	480	618	4652.1	0.019
Dichloromethane	480	623.8	4802.5	0.004
Acetone	480	623.7	4800.0	0.001
Acetonitrile	480	623.8	4802.5	0.001
Dimethylformamide	488	638.8	4837.4	0.001
Dimethyl sulfoxide	494			
Ethyl alcohol	497			
Methyl alcohol	493			

## Data Availability

The original contributions presented in the study are included in the article. Further inquiries can be directed at the corresponding author.

## Supplementary Materials

The following supporting information can be downloaded at: https://www.mdpi.com/article/10.3390/s26082411/s1, Figure S1: IR spectrum of 4-hydrazinyl-N-methyl-1,8-naphthalimide (**3**); Figure S2: ^1^H NMR spectrum of 4-hydrazinyl-N-methyl-1,8-naphthalimide (**3**) in DMSO; Figure S3: ^13^C NMR spectrum of 4-hydrazinyl-N-methyl-1,8-naphthalimide (**3**) in DMSO; Figure S4: HRMS spectrum of 4-hydrazinyl-N-methyl-1,8-naphthalimide (**3**); Figure S5: IR spectrum of 6-(2-(4-(dimethylamino)benzylidene)hydrazinyl)-2-methyl-1H-benzo[de]isoquinoline-1,3(2H)-dione (**5**); Figure S6: ^1^H NMR spectrum of 6-(2-(4-(dimethylamino)benzylidene)hydrazinyl)-2-methyl-1H-benzo[de]isoquinoline-1,3(2H)-dione (**5**) in DMSO; Figure S7: ^13^C NMR spectrum of 6-(2-(4-(dimethylamino)benzylidene)hydrazinyl)-2-methyl-1H-benzo[de]isoquinoline-1,3(2H)-dione (**5**) in DMSO; Figure S8: HRMS spectrum of 6-(2-(4-(dimethylamino)benzylidene)hydrazinyl)-2-methyl-1H-benzo[de]isoquinoline-1,3(2H)-dione (**5**).

## Abbreviations

The following abbreviations are used in this manuscript:

ICT	intramolecular charge transfer
PET	photoinduced electron transfer
TLC	thin-layer chromatography
UV/Vis	ultraviolet–visible spectroscopy
IR	infrared spectroscopy
^1^H NMR	proton nuclear magnetic resonance spectroscopy
^13^C NMR	^13^C nuclear magnetic resonance spectroscopy
HRMS	high-resolution mass spectra
DMF	dimethylformamide
