# Supplementary material for: New Naphthalimide Derivative as a Colorimetric and Fluorescent Probe for Detection of pH, Strong Bases and Volatile Acids
Source: Sensors (Basel). 2026 Apr 15;26(8):2411. doi: 10.3390/s26082411 (PMC13119930; doi:10.3390/s26082411)
Supplement: Supplementary file 1 [file sensors-26-02411-s001.zip › sensors-4229501-supplementary.pdf]

# New Naphthalimide Derivative as a Colorimetric and Fluorescent Probe for Detection of pH, Strong Bases and Volatile Acids

**Polya M. Miladinova**

Organic Synthesis Department, University of Chemical Technology and Metallurgy, 8 Kliment Ohridsky Blvd, 1797 Sofia, Bulgaria; ppolya@uctm.edu

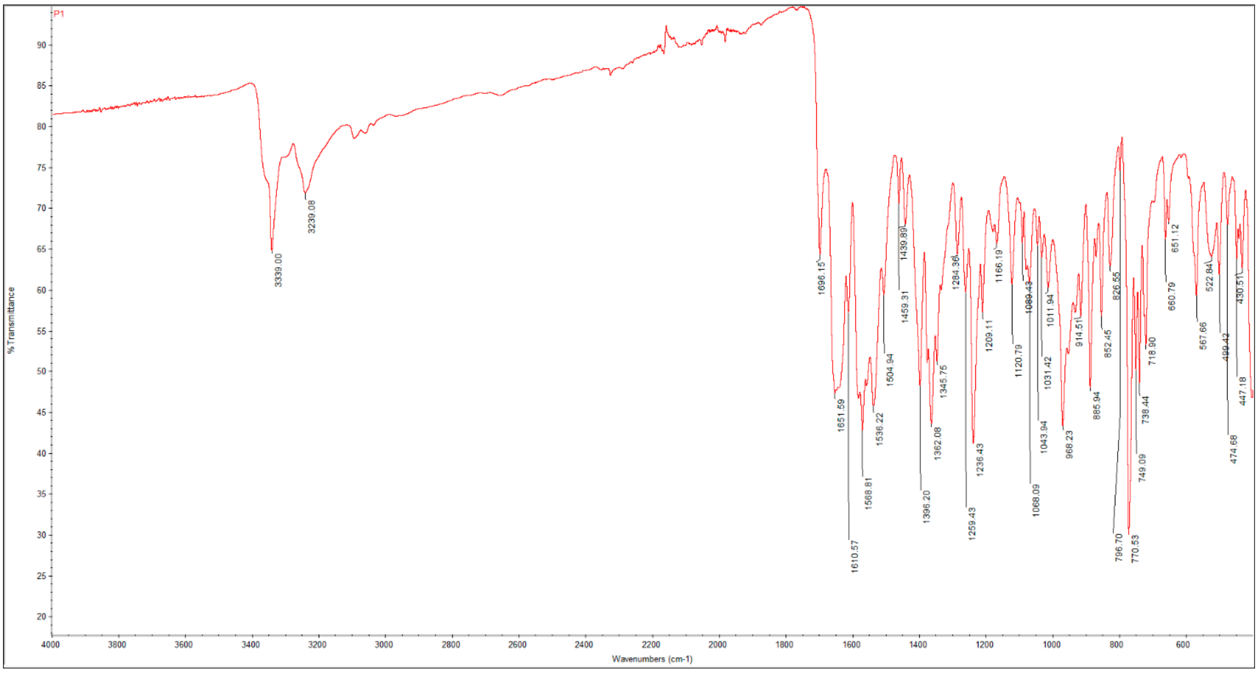


Figure S1: IR spectrum of 4-hydrazinyl-N-methyl-1,8-naphthalimide (3).

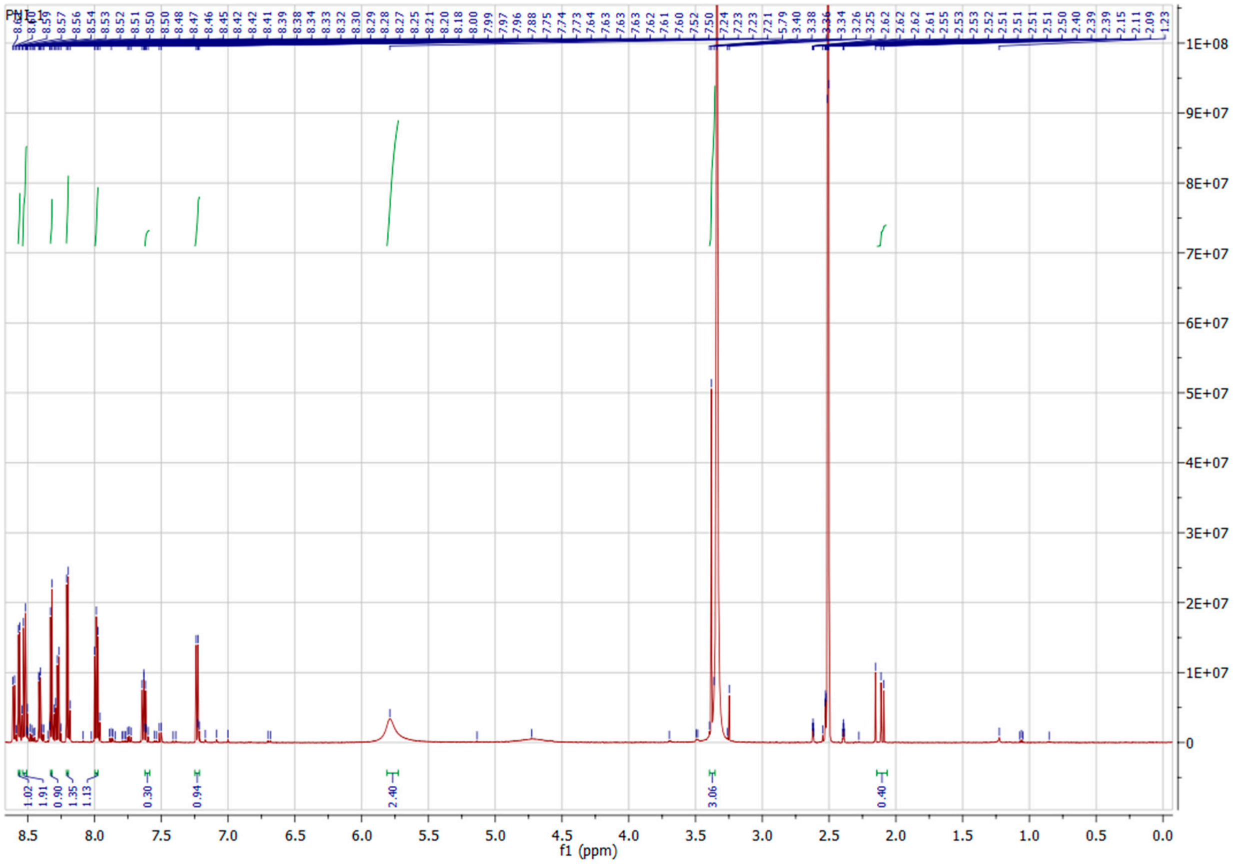


Figure S2: $^{1}$H NMR spectrum of 4-hydrazinyl-N-methyl-1,8-naphthalimide (3) in DMSO.

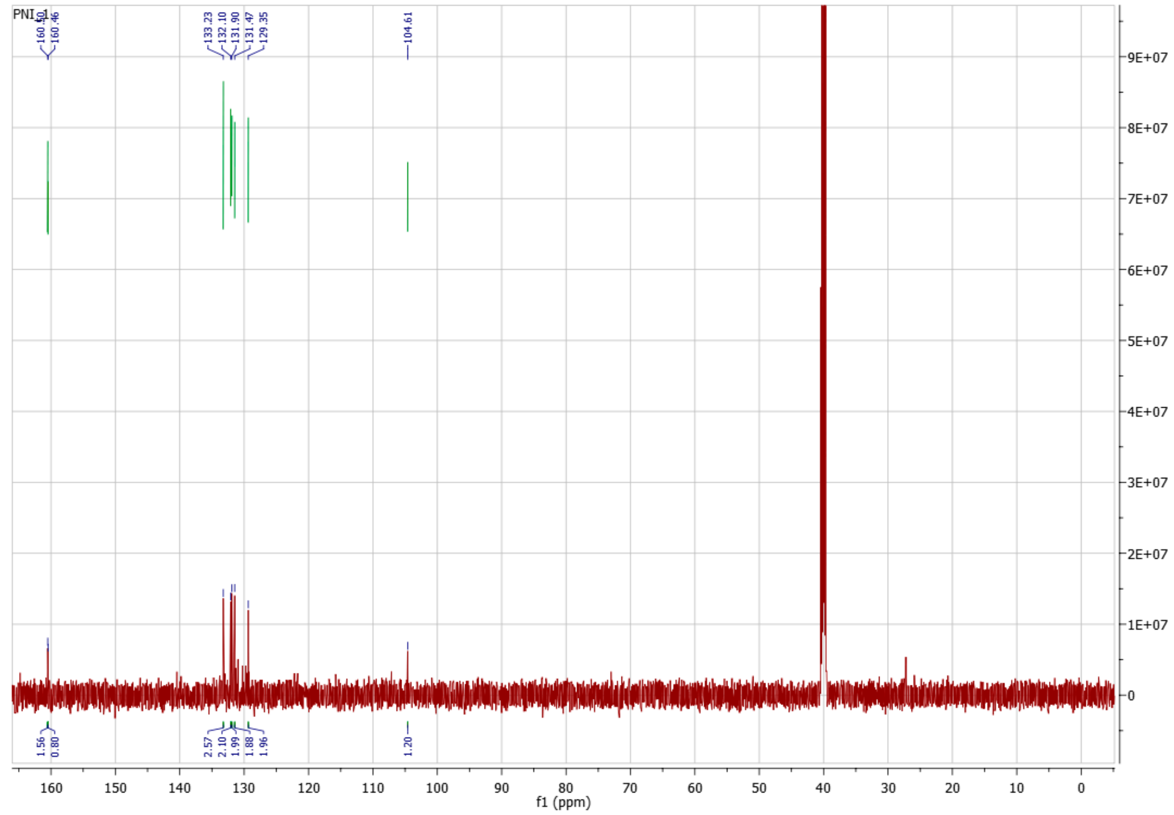


Figure S3: $^{13}C$ NMR spectrum of 4-hydrazinyl-N-methyl-1,8-naphthalimide (3) in DMSO.

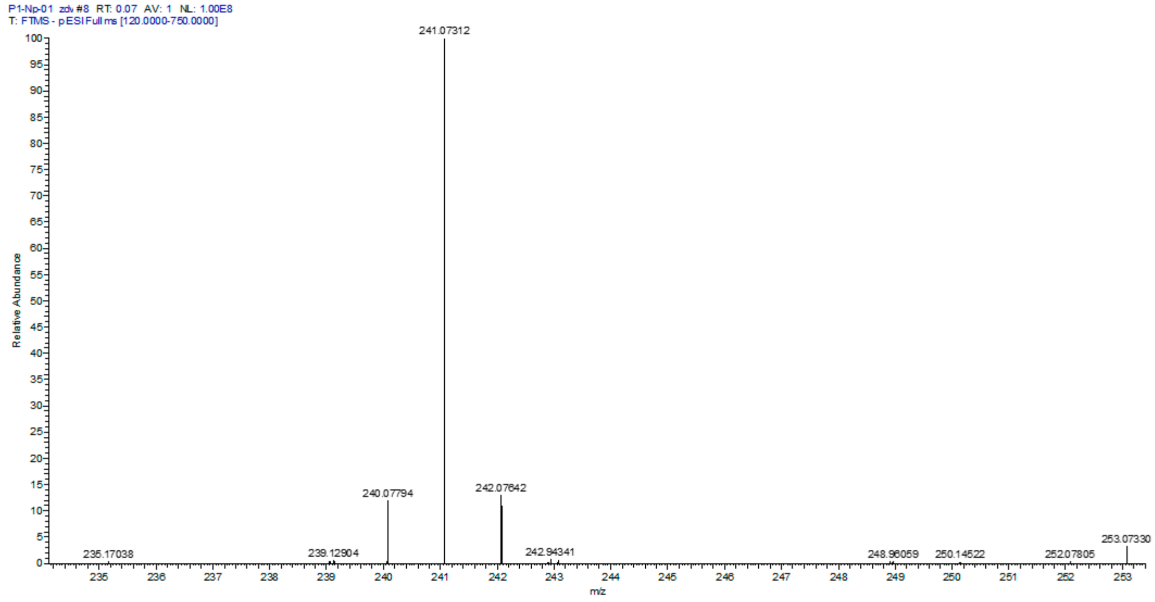


Figure S4: HRMS spectrum of 4-hydrazinyl-N-methyl-1,8-naphthalimide (3).

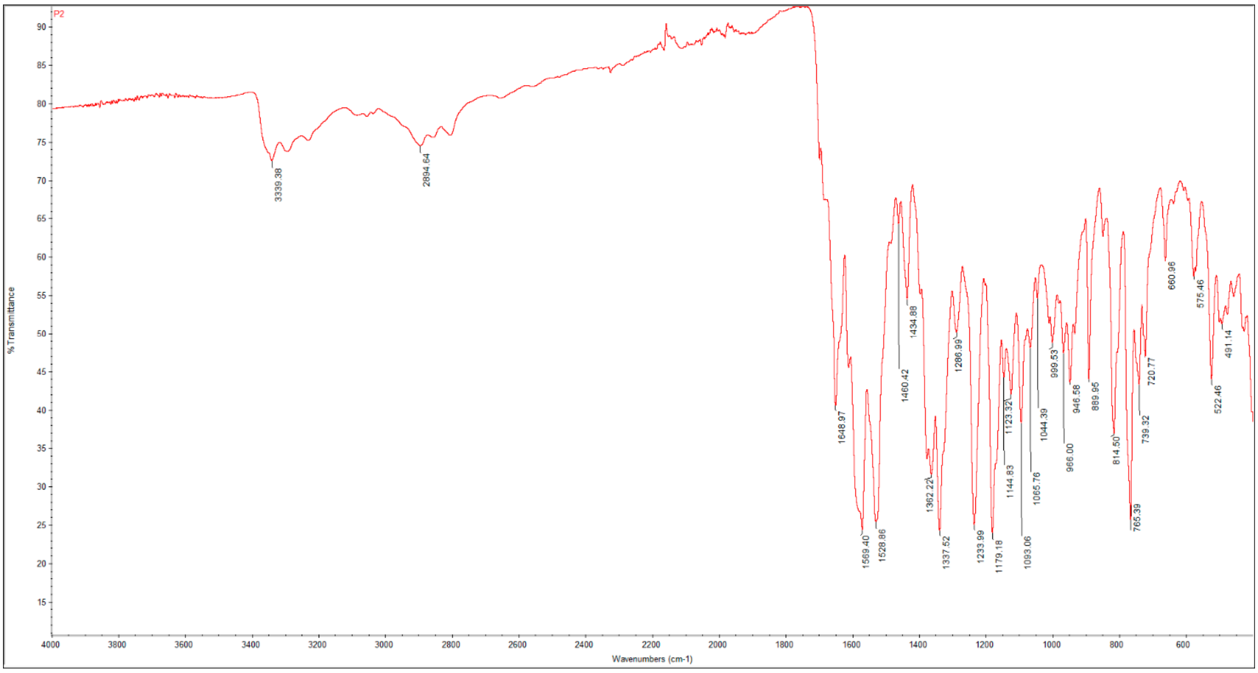


Figure S5: IR spectrum of 6-(2-(4-(dimethylamino)benzylidene)hydrazinyl)-2-methyl-1H-benzo[de]isoquinoline-1,3(2H)-dione (5).

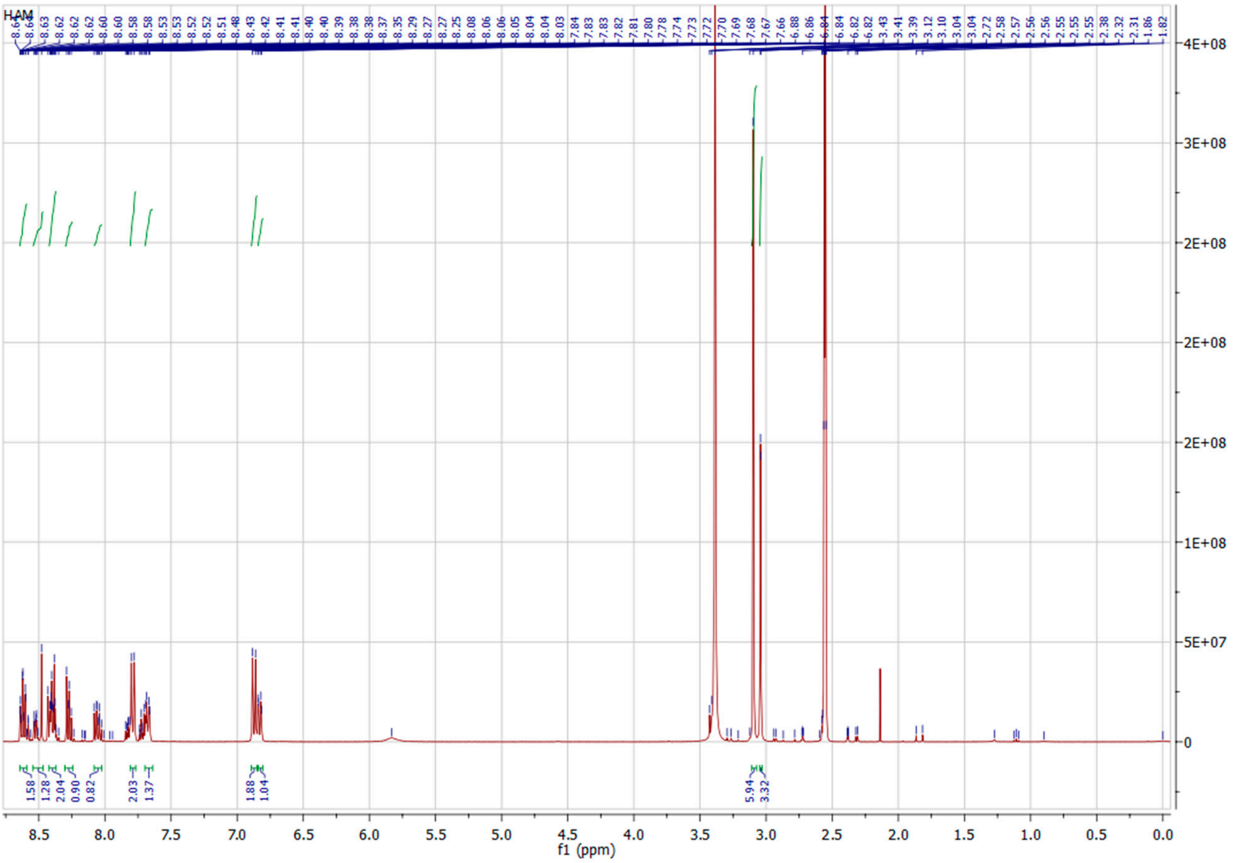


Figure S6: $^{1}$H NMR spectrum of 6-(2-(4-(dimethylamino)benzylidene)hydrazinyl)-2-methyl-1H-benzo[de]isoquinoline-1,3(2H)-dione (5) in DMSO.

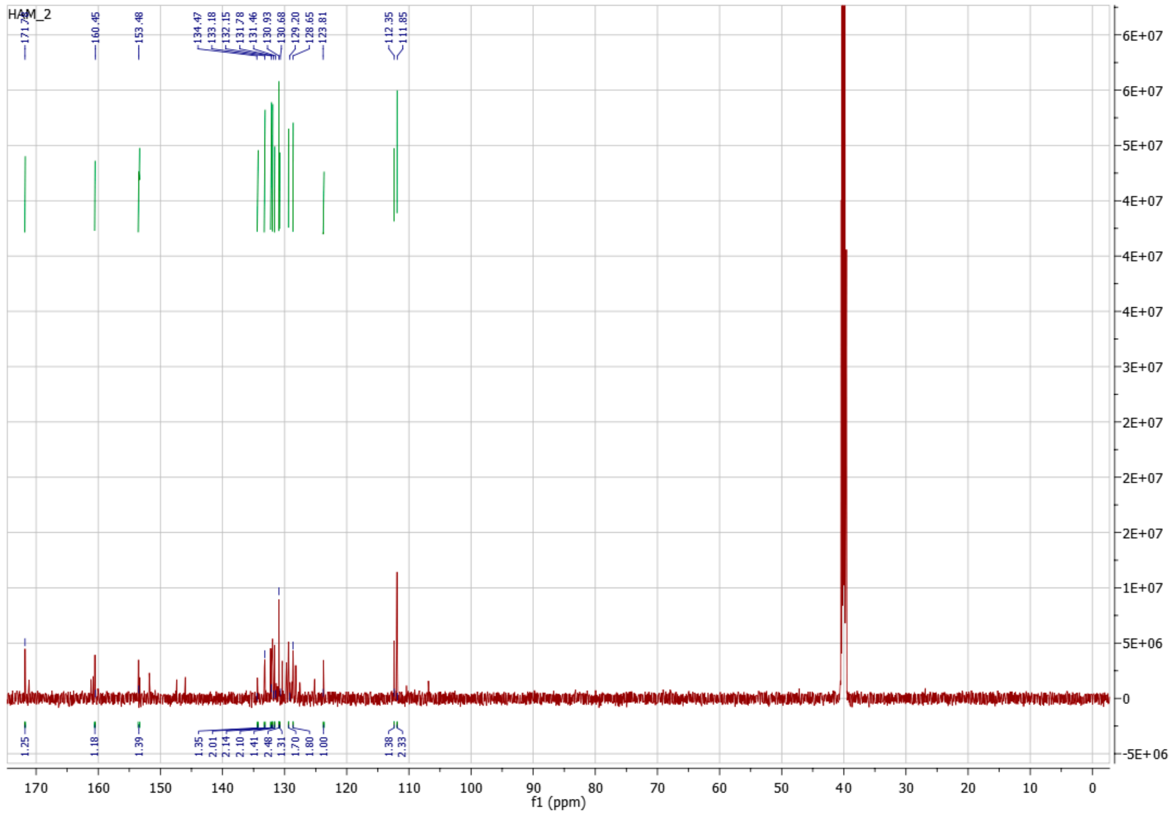


Figure S7: $^{13}$C NMR spectrum of 6-(2-(4-(dimethylamino)benzylidene)hydrazinyl)-2-methyl-1H-benzo[de]isoquinoline-1,3(2H)-dione (5) in DMSO.

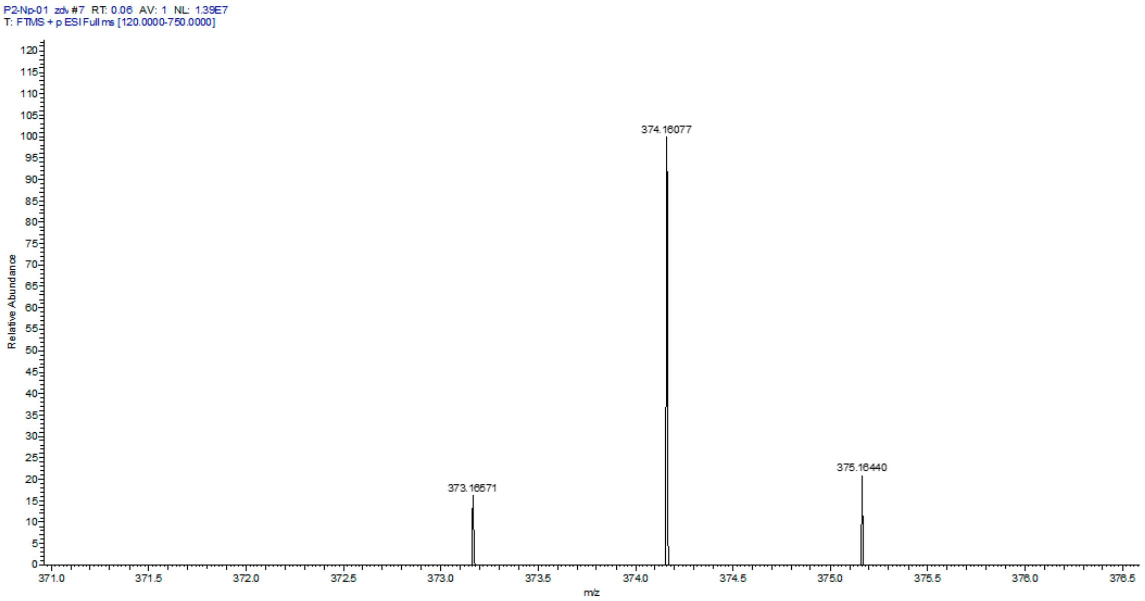


Figure S8: HRMS spectrum of 6-(2-(4-(dimethylamino)benzylidene)hydrazinyl)-2-methyl-1H-benzo[de]isoquinoline-1,3(2H)-dione (5).
